# Perforated mediastinal gastric volvulus: an uncommon complication of hiatal hernia

**DOI:** 10.1093/jscr/rjad102

**Published:** 2023-03-07

**Authors:** Mateus Henrique Schneider, Paola Solis-Pazmino, Pedro Maldonado, Mohamed Hamaoui

**Affiliations:** Surgery Department, Santa Casa de Misericórdia in Porto Alegre (SCMPA), Porto Alegre, RS 90035-074, Brazil; Surgery Department, Santa Casa de Misericórdia in Porto Alegre (SCMPA), Porto Alegre, RS 90035-074, Brazil; Surgery Department, Santa Casa de Misericórdia in Porto Alegre (SCMPA), Porto Alegre, RS 90035-074, Brazil; Surgery Department, Santa Casa de Misericórdia in Porto Alegre (SCMPA), Porto Alegre, RS 90035-074, Brazil

## Abstract

A woman in her 50s was admitted to the emergency department with a 3-day history of abdominal pain, mainly in the right hypochondrium, radiating to the back, associated with postprandial vomiting and dysphagia. The abdominal ultrasound study found no abnormalities. Laboratory tests showed increased C-reactive protein levels, creatinine and high white blood cell count without a left shift. Abdominal computed tomography scan exhibited mediastinal herniation, twist and perforation of the gastric fundus associated with air-fluid levels in the lower mediastinum. The patient underwent diagnostic laparoscopy requiring laparotomy conversion due to hemodynamic instability related to the pneumoperitoneum. During the intensive care unit (ICU) stay, thoracoscopy with pulmonary decortication was performed to treat complicated pleural effusion. After ICU and standard infirmary bed recovery, the patient was discharged from the hospital. This report illustrates a case of perforated gastric volvulus as the cause of nonspecific abdominal pain.

## INTRODUCTION

Gastric volvulus is a rare condition with an unknown incidence. It is defined as an abnormal rotation of the stomach over its axis that can occur acutely or chronically [1] and is potentially fatal [2]. Although the main presentation is intra-abdominal, it may appear as an intra-thoracic variation, which requires immediate diagnosis and treatment due to its less exuberant symptomatology and the risk of complications such as perforation and cardiorespiratory compromise [3, 4].

## CASE PRESENTATION

A woman in her 50s was admitted to the emergency department with a 3-day history of abdominal pain in the right upper quadrant radiating to the back, worsening in the last one, associated with postprandial vomiting and dysphagia. The patient had a referred medical history of hypothyroidism treated with 50 mg of levothyroxine once daily, gallstones, and a hiatal hernia, both asymptomatic. She had a surgical history of hysteroscopy for the excision of intrauterine polyps nine years before. At admission, the patient had stable and normal vital signs, was non-febrile and denied symptoms such as dyspnea, chest pain, urinary abnormalities and vaginal discharge. On physical exam, she reported pain on palpation in the right upper quadrant of the abdomen, without peritonism and negative Murphy’s sign. Cardiorespiratory auscultation was normal.

## INVESTIGATIONS

**Figure 1:**
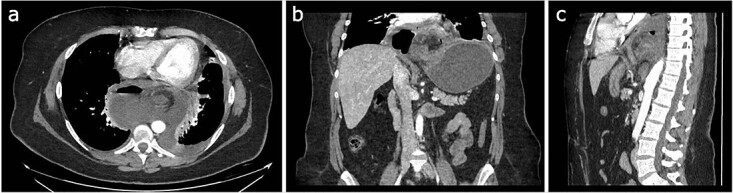
Different sections of the CT study demonstrate the mediastinal position of the gastritis volvulus. (**a**) Axial CT section of the abdomen showing the stomach body twisted inside the mediastinum. A hydro air level is seen at the right of the stomach body. (**b**) The coronal CT section of the thorax and the abdomen shows the twisting and dilatation of the reminiscent abdominal portion of the stomach. (**c**) Sagittal CT section of the thorax and the abdomen showing the posterior mediastinal position of the twisted stomach body.

Abdominal ultrasonography (USG) showed cholelithiasis without cholecystitis or other anomalies. However, a contrast-enhanced CT exhibited distension of the gastric chamber, with inversion of the positions of the pylorus and the esophagogastric junction, and free intraabdominal air/features suggesting perforation (Fig. 1). Laboratory testing revealed an elevated white blood cell count of 22 K/uL and a protein C-reactive of 257. Electrolytes were within normal limits. Amylase and lipase were normal.

## DIFFERENTIAL DIAGNOSIS

Although the leading risk factor of the patient was hiatal hernia, the clinical presentation was nonspecific. Additionally, other conditions that result in acute abdominal pain had a higher index of suspicions, such as acute cholecystitis, pancreatitis and peptic ulcer disease.

Cholecystitis was ruled out due to the patient not experiencing a fever, Murphy’s sign and the abdominal USG did not find any abnormalities.

Pancreatitis was rejected because amylase and lipase levels were normal.

Peptic ulcer disease was least suspected because the patient did not show burning stomach pain, nausea or heartburn.

## TREATMENT

**Figure 2:**
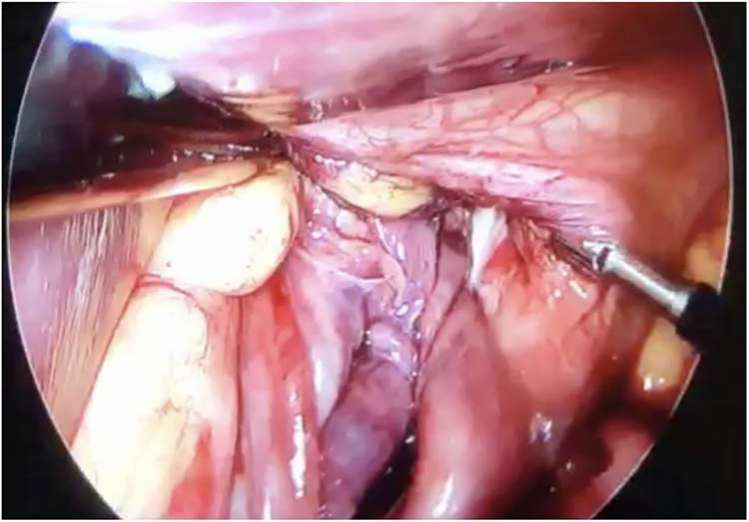
Twisted stomach body protruding through the diaphragmatic pillars, laparoscopic view.

**Figure 3:**
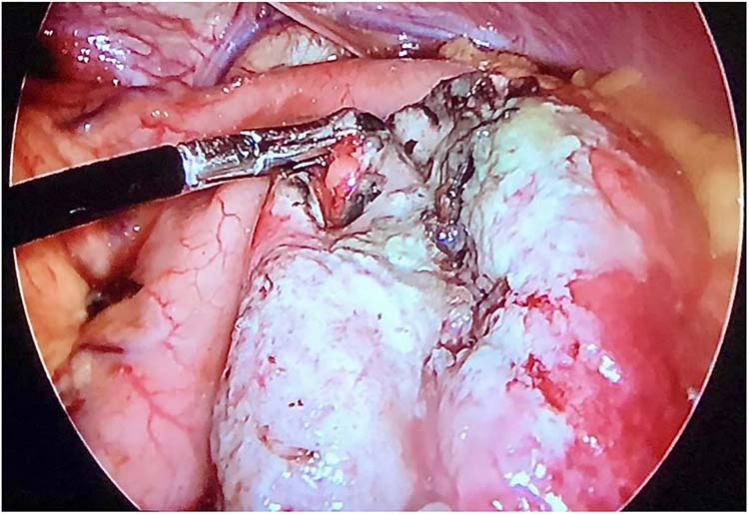
Laparoscopic view demonstrating an approximately 1.5-cm lesion at the level of the greater curvature of the stomach.

**Figure 4:**
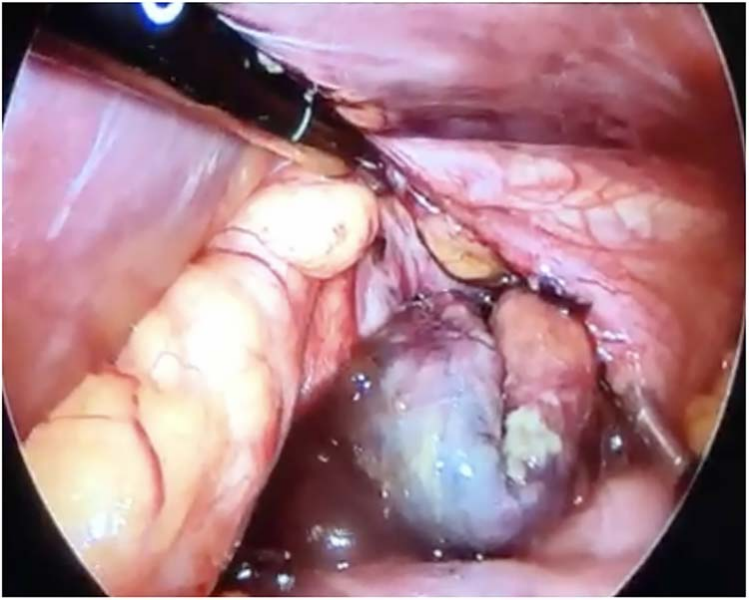
Laparoscopic view of the necrotic hernial sac with food scraps inside.

Initial treatment included intravenous crystalloid fluid repositioning, pain management, and antibiotic therapy. After diagnosing perforated gastric volvulus on a computed tomography (CT) scan, a diagnostic laparoscopy was performed. A hiatal hernia was identified with protrusion and twisting of the gastric body (Fig. 2). As the stomach body was reduced back to the abdominal cavity, a 1.5-cm perforation at the level of the greater curvature was detected. It was primarily sutured and covered with an omental patch (Fig. 3). The ischemic hernia sac containing the stomach was filled with food scraps and resected (Fig. 4). Laparotomy conversion was necessary due to hemodynamic instability attributable to the laparoscopic pneumoperitoneum. A 180° anterior fundoplication of the stomach (Dor’s Technic) associated with diaphragmatic raffia was performed. Gastric air leak testing was negative and intraoperative endoscopy showed no other defects on the gastric wall.

## OUTCOME AND FOLLOW-UP

Postoperatively, the patient was stable and with good recovery. However, two weeks after, the patient presented a pulmonary thromboembolism and aspiration pneumonia during an extubating attempt in the ICU. The patient was treated with anticoagulation, antibiotic and pulmonary physical therapy. A week later, the patient presented elevated inflammatory laboratory parameters and an unsatisfactory ventilatory pattern. A complicated pleural effusion was detected on a thorax CT scan. Thoracoscopic decortication of the left lung was successfully performed. After ICU release and standard infirmary bed recovery, the patient was discharged from the hospital. Presently, she is attending regular medical consultation follow-ups every three months.

## DISCUSSION

Gastric volvulus is a rare disease uncommonly considered the initial underlying cause of abdominal pain and can have a missed or delayed diagnosis and treatment [2].

Although 70% of cases report Borchardt’s triad (vomiting, epigastric pain and inability to pass a nasogastric tube), the clinic was unspecific and broad in this patient. It may be because sudden twisting of the volvulus did not present a complete obstruction or strangulation [5].

Also, symptoms depend upon the type of volvulus. The most common (59%) is the organoaxial gastric volvulus which results from the rotation of the stomach around its long axis [2]. The second type is the mesenteroaxial, caused by torsion around the short stomach axis. The peak of organo-axial volvulus incidence is in the fifth decade, whereas the mesenteroaxial volvulus has been reported in neonates, infants, and young children. Our case is rare because it is a mesenteroaxial volvulus in a middle-aged woman with non-specific symptoms [3].

When the clinical presentation is uncommon, the diagnosis is complex and based on image investigation. It can be chest radiography, the standard gold test or the upper gastrointestinal tract fluoroscopy. However, CT scans provide a more accurate diagnosis with specific anatomical details. Our patient underwent a CT scan documenting a mesenteroaxial volvulus and a hiatal hernia [6].

The definitive treatment is surgical. The laparoscopic surgical approach allows the repair of the volvulus and decreases the chance of recurrence through diaphragmatic hernia correction [7].
